# Should routine neonatal circumcision be a police to prevent penile cancer? | Opinion: Yes

**DOI:** 10.1590/S1677-5538.IBJU.2017.01.03

**Published:** 2017

**Authors:** Antonio Augusto Ornellas, Paulo Ornellas

**Affiliations:** 1 Departamento de Urologia, Instituto Nacional do Câncer do Brasil (INCA);; 2 Departamento de Urologia Hospital Mário Kröeff, Rio de Janeiro, Brasil;; 3 Departamentos de Urologia, Hospital Souza Aguiar Hospital, Departamento de Patologia, Laboratório de Biometria Circulante;; 4 Programa de Pós-Graduação em Ciências Médicas (PGCM), Universidade Estadual Rio de Janeiro State, Rio de Janeiro, Brasil

**Keywords:** prevention and control [Subheading], Circumcision, Male, Penile Neoplasms, Phimosis

This theme is controversial because no major medical organization recommends universal neonatal circumcision and no major medical organization calls for banning it either. The argument that this procedure must be kept within the purview of medical professionals is found across all major medical organizations. In addition, the organizations advise medical professionals to yield to some degree to parents’ preferences, commonly based in cultural or religious views, in the decision to agree to circumcise (1). Circumcision may be used to treat pathological phimosis, refractory balanoposthitis and chronic, recurrent urinary tract infections (2, 3). Circumcision is contraindicated in infants with certain genital structure abnormalities, such as a misplaced urethral opening (as in hypospadias and epispadias), curvature of the head of the penis (chordee), or ambiguous genitalia, because the foreskin may be needed for reconstructive surgery. Circumcision is contraindicated in premature infants and those who are not clinically stable and in good health (3-5). If an individual, child or adult, is known to have or has a family history of serious bleeding disorders (hemophilia), it is recommended that the blood be checked for normal coagulation properties before the procedure is attempted (3, 5). A 2010 review of literature worldwide found circumcisions performed by medical providers to have a median complication rate of 1.5% for newborns and 6% for older children, with few cases of severe complications (6). Bleeding, infection and the removal of either too much or too little foreskin are the most common complications cited (6). Complication rates are higher when the procedure is performed by an inexperienced operator, in unsterile conditions, or when the child is at an older age (6). Circumcision does not appear to have a negative impact on sexual function (7).

The practice of neonatal circumcision exerts a protective factor avoiding the genesis of penile cancer. While the presence of phimosis is a strong risk factor for penile cancer, neonatal circumcision appears to be a protective factor (8, 9). The incidence of penile cancer in the Jewish population, where the practice of neonatal circumcision is universally practiced, approaches zero. There are only 9 reports of penile cancer in circumcised Jews in the neonatal period, reported in the world literature. Interestingly, our group had the opportunity to treat an Israeli patient, of Jewish religion, who underwent neonatal circumcision and had an advanced penile tumor (10). The incidence rate of penile cancer in India where circumcision is not performed routinely, is 3.32 / 100,000 inhabitants, compared with rates close to zero found in Jews born in Israel. In countries of Muslim religion, where circumcision is performed in infancy, outside the neonatal period, there is an increase in the incidence of this neoplasia by up to 3 times (11). Several studies observed an increased risk for invasive penile cancer among men not circumcised in childhood (9, 12). The presence of a foreskin do not increase the risk on penile cancer however the presence of phimosis in men with penile carcinoma is high, ranging from 44% to 85% (8). Phimosis leads invariably to retention of smegma resulting in conditions of chronic irritation with or without bacterial inflammation of the prepuce and the glans. Smegma is a whitish film found under the foreskin of uncircumcised males. It contains bacteria, other microorganisms, dead skin cells, mucous, and other components. Smegma may cause chronic inflammation and recurrent infections that lead to preputial adhesions and phimosis. Substantial increased relative risk for penile cancer was recorded (up to 65-fold) among males with phimosis (8, 9).

Infection by high-risk HPV group is probably the major cause of anogenital cancers. High transmission potential with a low impact on herd immunity means extensive vaccination would be required to substantially reduce the incidence of cancer of the cervix and penis caused by high-risk HPV types. Further, vaccination of males against HPV appears to represent an expensive measure for prevention of penile cancer, particularly when one considers that high-risk HPV is present in only half of penile cancers. HPV vaccination of males should nevertheless help reduce cervical, anal and perhaps oropharyngeal cancers.

On the other hand, lack of circumcision is a risk factor for phimosis and balanitis which themselves are risk factors for penile cancer. This would explain why invasive penile cancer is rare in circumcised men

Circumcised men are consistently less likely than uncircumcised men to have HPV infection at the glans/corona and urethra. Several studies showed that male circumcision is associated with an overall reduction in the prevalence of genital HPV infection in men (13-16). These site-specific effects possibly occur because the foreskin provides a suitable environment around the glans for HPV infection (13) and HPV type-specific concordance has been shown between the glans/corona and foreskin in uncircumcised men that possibly reflects simultaneous infection or autoinoculation (17). Thereby, male circumcision reduces the risk of HPV infection among men and consequently reduces the exposure of women to high-risk HPV. It explains why women with circumcised partners are at lesser risk of cervical cancer. Hence, the observed evidence for a protective effect of male circumcision on cervical HPV infection has prompted the suggestion that male circumcision could be considered a major intervention measure to prevent the incidence of both diseases (18). IARC study (19) found strong epidemiological evidence that male circumcision is associated with a reduced risk of genital HPV infection in men and with a reduced risk of cervical cancer in women, notably among women with high-risk partners. Male circumcision may supplant HPV vaccines in protecting against other different genotypes of HPV and would be a tangible tool to reduce female genital infections.

In recent study, our group found HPV in 46.66% of our patients with phimosis, of whom 50% had high risk HPV genotypes. Of asymptomatic cases 16.36% were HPV positive but only 1 sample showed high risk HPV. We detected a significantly high rate of HPV genital infection in patients presenting with phimosis compared with asymptomatic men (p=0.00167). The prevalence of high risk HPV genotypes in patients with phimosis was also statistically significant (p=0.0004). We found a robust association between phimosis and the genital HPV prevalence in men and a significant frequency of high risk HPV. However, more studies are needed to adequately assess the effect of male circumcision on the acquisition and clearance of HPV infections. The focus of the treatment with vaccination or circumcision should be men in early age range (20). Taking into account the literature data and the limitations to perform the surgery, cited above, neonatal circumcision would be the best procedure to prevent penile cancer.
